# Ringed fluorodeoxyglucose uptake predicted poor prognosis after resection of pulmonary pleomorphic carcinoma

**DOI:** 10.1186/s13019-022-01799-6

**Published:** 2022-03-21

**Authors:** Yutaka Shishido, Akihiro Aoyama, Shigeo Hara, Yuki Sato, Keisuke Tomii, Hiroshi Hamakawa, Yutaka Takahashi

**Affiliations:** 1 Department of General Thoracic Surgery, Kobe City Medical Center General Hospital, 2-2-1, Minatojimaminamimachi, Chuo-ku, Kobe-city, Hyogo 6500047 Japan; 2 Department of Diagnostic Pathology, Kobe City Medical Center General Hospital, Kobe, Hyogo Japan; 3 Department of Respiratory Medicine, Kobe City Medical Center General Hospital, Kobe, Hyogo Japan

**Keywords:** Lung cancer, Pleomorphic carcinoma, Prognostic factor, PET-CT, Programmed cell death ligand 1

## Abstract

**Background:**

Pulmonary pleomorphic carcinoma (PPC) is a relatively rare and poorly differentiated non-small cell carcinoma. This study aimed to investigate the clinicopathological features including programmed cell death ligand 1 (PD-L1) expression status in patients with PPC who underwent curative resection.

**Methods:**

We retrospectively studied 29 consecutive patients who had undergone anatomical lung resections for PPC. Perioperative and pathological variables, including radiological findings, were investigated to define prognostic factors.

**Results:**

Overall survival (OS) rates were 71.8% at 1 year and 60.0% at 5 years. Disease-free survival (DFS) rates were 54.8% at 1 year and 43.6% at 5 years. Univariate analysis revealed that ringed fluorodeoxyglucose (FDG) uptake on positron emission tomography/computed tomography (PET/CT) (*p* = 0.003), a cavity in the tumor on CT (*p* = 0.004), and tumor size (> 40 mm) (*p* = 0.014) were poor prognostic factors for OS. Regarding DFS, ringed FDG uptake (*p* = 0.002), a cavity on CT (*p* < 0.001), tumor size (*p* = 0.007), and pleural invasion (*p* = 0.014) were poor prognostic factors. PD-L1 expression was not a prognostic factor.

**Conclusion:**

This study showed for the first time that ringed FDG uptake on PET/CT is a poor prognostic factor of PPC. PD-L1 expression status was not related to the prognosis.

*Trial registration* The study was approved by the Kobe City Medical Center General Hospital’s ethics board (No. 20112) on August 20, 2020.

## Background

Pulmonary pleomorphic carcinoma (PPC) is a rare epithelial malignant tumor that accounts for approximately 0.4% of all lung cancers [1]. The 2015 World Health Organization (WHO) classification defines PPC as a poorly differentiated non-small cell carcinoma that contains at least 10% spindle and/or giant cells or a carcinoma consisting only of spindle and giant cells [2]. Compared to other histological types of non-small-cell lung cancers (NSCLCs), PPC is associated with a more aggressive clinical course and worse prognosis despite complete resection and adjuvant chemotherapy [3, 4]. Given the current situation, we need to seek novel treatment approaches, such as the administration of immune checkpoint inhibitors (ICIs). However, due to its rarity, there is no consensus about the clinicopathological characteristics of PCP and clinical implications of a novel biomarker, programmed cell death ligand 1 (PD-L1) expression.

In this study, we retrospectively investigated patients with PPC who were surgically treated in our hospital and tried to identify the prognostic factors and the clinicopathological features associated with the disease.

## Methods

**Fig. 1:**
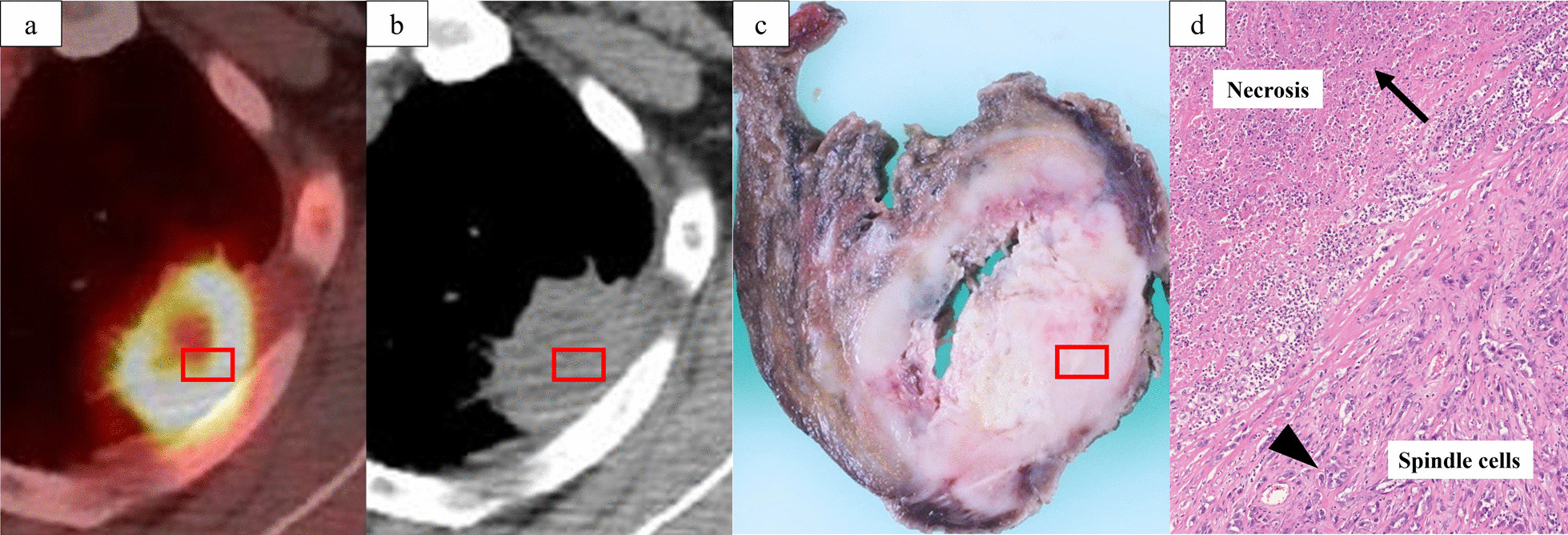
A 72-year-old male patient with pT3N0M0 pulmonary pleomorphic carcinoma. **a** Fused PET/CT image obtained before surgery shows ringed FDG uptake in the tumor (**b**) Axial non-contrast CT image on the same day shows peripheral solid mass without cavity formation. **c** The gross specimen shows a mainly solid tumor with a crescent-like cavity. **d** Microscopic examination of areas in the red squares shows necrosis on the upper left side (arrow) and spindle cell on the lower right side (triangle) (Hematoxylin and eosin staining, × 100)

We retrospectively reviewed the data of 29 PPCs (1.5%) from a total of 1903 primary lung cancer patients surgically treated at Kobe City Medical Center General Hospital between January 2006 and April 2020. Clinical information, including age, sex, smoking habits, and clinical stage at presentation, was obtained from the medical records. All patients underwent chest computed tomography (CT) before the surgery, and we evaluated tumor size, lymph nodes, the presence of the cavity in the tumor, and the location of the tumor. A peripheral tumor was defined as being located in the outer third of the lung field. Positron emission tomography/CT (PET/CT) and brain magnetic resonance imaging (MRI) were also conducted to evaluate lymph nodes and distant metastases. We also addressed the ringed fluorodeoxyglucose (FDG) uptake on PET/CT, which is defined as donut-like FDG accumulation exclusively at the rim of a tumor regardless of whether or not a cavity is formed in the tumor (Fig. 1a). All pathological materials were reviewed by expert pathologists at our hospital. The diagnosis of PCP was obtained by light microscopy findings and immunohistochemical examinations according to the 2015 WHO classification [2]. Pathological lymph node status, lymphovascular invasion, and pleural invasion were also examined. The study was approved by the Kobe City Medical Center General Hospital’s ethics board (No. 20112) on August 20, 2020. Written informed consent for this study was obtained from patients who were alive at data collection.

Programmed cell death ligand 1 (PD-L1) expression was assessed on 4-μm-thick sections of formalin-fixed paraffin-embedded tissue blocks, using anti-PD-L1 antibody (clone E1L3N, 1:200, Cell Signaling Technology, Danvers, MA). We evaluated the PD-L1 tumor proportion score (TPS), and the PD-L1 status was considered positive when TPS ≥ 5% [5].

Overall survival (OS) and disease-free survival (DFS) were assessed using the Kaplan–Meier method, and univariate analyses were performed using the log-rank test. The correlations between PD-L1 expression and clinicopathological characteristics were evaluated using Fisher’s exact test for categorical variables and Student’s t-test for numerical variables. Data were analyzed statistically using EZR (Saitama Medical Center, Jichi Medical University, Saitama, Japan), which is a graphical user interface for R (The R Foundation for Statistical Computing, Vienna, Austria) [6]. All *P* values were two-sided, and differences were considered statistically significant at *P* < 0.05.

## Results

All the clinicopathological data of the 29 patients included in this study are shown in Table 1. There were 20 male (69.0%) and 9 female (31.0%) patients. The median age was 70 years (range: 44–88 years). Twenty-two patients (75.9%) had a smoking history, and 3 (10.3%) were diagnosed with PPC before surgery. Regarding the CT findings, the tumor was located in the peripheral field of the lung in 19 cases (65.5%). The cavity in the tumor was found in 8 patients (27.6%). FDG uptake was observed in tumors of all 27 patients who preoperatively underwent PET/CT, and among them, ringed uptake was found in 12 patients (44.4%). In 5 cases, CT showed no cavity, but fused PET/CT showed ringed FDG uptake, with a representative case shown in Fig. 1. No apparent cavity was macroscopically noted, but necrosis in the central site and tumor cells in the peripheral site were observed in correspondence with the PET/CT image (Fig. 1). The surgical procedures included 25 lobectomies, 1 bilobectomy, and 3 pneumonectomies. The pathological stage was I in 8 patients, II in 13, III in 7, and IV in 1 who had been surgically treated for a single brain metastasis (8th edition of the Union for International Cancer Control [UICC]) [7]. The median tumor size was 38 mm (range: 15–92 mm). Nodal status was classified as pN0 in 22 patients (75.9%), pN1 in 4 patients (13.8%), and pN2 in 3 patients (10.3%). Lymphatic invasion was observed in 4 patients (13.8%) and vascular invasion in 15 patients (51.7%). Pleural invasion was observed in 16 patients (55.1%). PD-L1 expression (TPS ≥ 5%) was observed in 20 patients (69.0%). Platinum-based adjuvant chemotherapy was administered to 8 patients (27.6%) who were at pStages II and III.

**Table 1:** Patients’ preoperative, pathological, and postoperative data

Case	Age	Sex	B.I	Cavity on CT	Ringed FDG uptake	Tumor size (mm)	p-TNM	p-Stage	Pl	Ly	V	TPS (%)	DFS (m.o.)	OS (m.o.)	Outcome
1	56	m	900	No	No	34	T2N0M0	I	0	0	0	30	91	91	Alive
2	70	f	1000	No	Yes	42	T2N0M0	II	0	0	0	0	54	54	Death
3	69	f	0	No	No	32	T2N1M0	II	3	0	1	0	52	52	Alive
4	62	m	1180	No	No	20	T3N0M0	II	3	0	0	70	104	104	Alive
5	67	m	1000	No	No	25	T3N0M0	II	0	0	0	90	48	48	Alive
6	54	m	700	No	Not available	38	T2N1M0	II	2	0	1	40	70	70	Alive
7	55	m	600	No	Yes	70	T3N0M0	II	1	0	1	30	48	48	Alive
8	70	m	225	No	No	42	T2N0M0	II	0	0	0	2–3	46	46	Alive
9	72	f	0	Yes	Yes	43	T2N0M0	II	1	0	1	0	50	50	Alive
10	71	m	0	No	No	22	T1N0M0	I	0	0	0	10	49	49	Alive
11	71	f	0	No	No	15	T1N0M0	I	0	0	0	0	76	76	Alive
12	69	f	0	No	No	26	T1N0M0	I	0	0	0	0	99	99	Alive
13	78	m	1600	No	No	25	T1N2M0	III	0	0	0	90	34	34	Alive
14	62	m	0	No	No	22	T2N2M0	III	1	0	1	0	35	94	Alive
15	70	f	1600	No	No	15	T1N0M0	I	0	0	1	80	77	77	Alive
16	80	m	800	Yes	Yes	92	T4N0M0	III	0	0	0	90	3	69	Death
17	65	m	940	No	No	60	T3N0M0	II	2	0	1	50	5	41	Alive
18	88	m	1000	Yes	Yes	45	T2N0M0	II	2	0	0	70	3	3	Death
19	75	m	1100	No	No	38	T3N1M0	III	3	0	1	70	3	3	Death
20	44	m	1000	Yes	Yes	90	T4N0M1	IV	1	1	1	90	1	6	Death
21	74	m	500	No	Yes	85	T4N0M0	III	1	0	0	2–3	1	3	Death
22	49	m	600	No	Yes	24	T1N0M0	I	0	1	1	0	6	7	Death
23	80	f	0	No	No	40	T2N0M0	I	1	0	0	5	12	38	Death
24	59	f	800	Yes	Not available	55	T3N0M0	II	3	0	0	80	0	3	Death
25	78	m	2000	Yes	Yes	35	T2N2M0	III	0	0	1	80	1	1	Death
26	72	m	1560	No	Yes	41	T3N0M0	II	3	0	1	90	3	11	Alive
27	73	m	1000	Yes	Yes	42	T2N0M0	II	2	0	1	60	4	4	Death
28	72	f	880	No	No	25	T3N1M0	III	0	1	1	100	6	6	Alive
29	76	m	2320	Yes	Yes	25	T2N0M0	I	2	1	1	10	3	5	Alive

*BI* Brinkman index, *CT* computed tomography, *FDG* fluorodeoxyglucose, *Pl* plerual invasion, *Ly* lymphative invasion, *V* vascular invasion, *TPS* tumor proportion score, *DFS* disease-free survival, *OS* overall survival, *m.o.* months, *m* male, *f* female

**Fig. 2:**
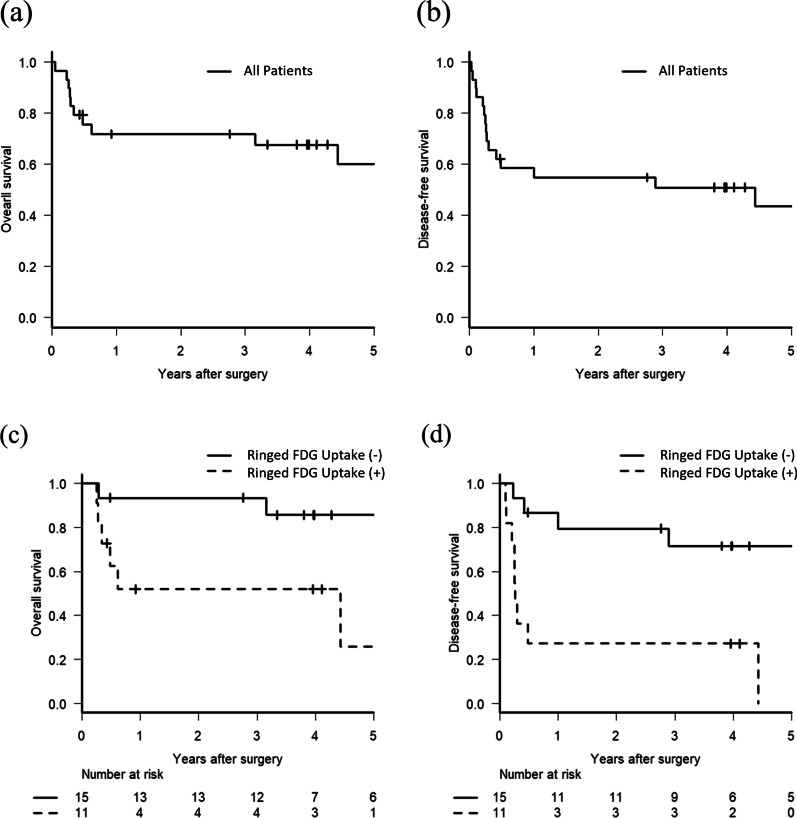
Kaplan–Meier curves of overall survival (**a**) and disease-free survival (**b**) for patients with surgically resected pleomorphic carcinoma, and Kaplan–Meier curves of overall survival (**c**) and disease-free survival (**d**) according to the ringed FDG uptake on PET/CT

The mean follow-up time was 4.3 years (range: 0.1–8.7 years). One patient died of acute exacerbation of underlying interstitial pneumonia 18 days after surgery, 9 patients of PPC recurrence, and 1 of a second lung cancer. During the follow-up period, PPC recurrence was observed in 13 patients (44.8%), mediastinal lymph node metastasis in 1 patient and distant recurrence in 12, including malignant pleural effusion in 2 patients. The OS rates after pulmonary resection were 71.8% at 1 year and 60.0% at 5 years (Fig. 2a). The DFS rates after pulmonary resection were 54.8% at 1 year and 43.6% at 5 years (Fig. 2b), indicating that most relapses were observed within one year of surgery. The results of the univariate analysis for long-term prognosis are summarized in Table 2. We excluded 1 patient who died of acute exacerbation of interstitial pneumonia shortly after surgery. Ringed FDG uptake (*p* = 0.003; Fig. 2c), a cavity on CT (*p* = 0.004), and tumor size (> 40 mm) (*p* = 0.014) were significant poor prognostic factors for OS. Regarding DFS, ringed FDG uptake (*p* = 0.002; Fig. 2d), a cavity on CT (*p* < 0.001), tumor size (> 40 mm) (*p* = 0.007), and pleural invasion (*p* = 0.014) were significant poor prognostic factors. The distribution of clinicopathological characteristics according to PD-L1 expression is shown in Table 3, which shows that PD-L1 expression was significantly correlated only with smoking history (*p* = 0.016).

**Table 2:** Univariate analysis of prognostic factors associated with disease-free survival and overall survival

Characteristic, risk factor	Disease-free survival	Overall survival
	*p* value	*p* value
Age, > 70 y	0.118	0.128
Sex, male	0.193	0.742
Smoking, former or current	0.137	0.140
Cavity on CT	< 0.001	0.004
Ringed FDG uptake on PET/CT	0.002	0.003
Tumor size, > 40 mm	0.007	0.014
pN classification, N > 0	0.445	0.374
Lymphatic invasion	0.104	0.222
Vascular invasion	0.434	0.652
Pleural invasion	0.014	0.157
PD-L1 expression, TPS ≥ 5%	0.438	0.661

*CT* chest computed tomography, *FDG* fluorodeoxyglucose, *PET/CT* positron emission tomography/CT, *PD-L1* programmed cell death ligand 1

**Table 3:** Distribution of clinicopathological characteristics according to programmed cell death ligand 1 expression

Characteristics	All patients (%)	PD-L1 ≥ 5% (%)	PD-L1 < 5% (%)	*p* value
*Age (years)*				
Median [range]	70 [44–88]	72 [44–88]	70 [49–72]	0.561
*Sex*				
Male	20 (69.0)	16 (80)	4 (44.4)	0.088
Female	9 (31.0)	4 (20)	5 (55.6)	
*Smoking status*				
Never	7 (24.1)	2 (10)	5 (55.6)	0.016
Former/current	22 (75.9)	18 (90)	4 (44.4)	
*Cavity on CT*				
Absent	21 (72.4)	13 (65)	8 (89)	0.371
Present	8 (27.6)	7 (35)	1 (11)	
*Ringed FDG uptake on PET/CT*				
Absent	15 (55.6)	10 (55.6)	5 (55.6)	1
Present	12 (44.4)	8 (44.4)	4 (44.4)	
*Tumor size (mm)*				
Median [range]	38 [15–92]	38 [15–92]	32 [15–85]	0.561
*pT classification*				
T1	6 (20.7)	3 (15.8)	3 (30)	0.072
T2	12 (41.4)	6 (31.6)	6 (60)	
T3	8 (27.6)	8 (42.1)	0 (0)	
T4	3 (10.3)	2 (10.5)	1 (10)	
*pN classification*				
N0	22 (75.9)	15 (75)	7 (77.8)	1
N1	4 (13.8)	3 (15)	1 (11.1)	
N2	3 (10.3)	2 (10)	1 (11.1)	
*Pathological stage*				
I	8 (27.6)	5 (25)	3 (33.3)	1
II	13 (44.8)	9 (45)	4 (44.4)	
III	7 (24.1)	5 (25)	2 (22.2)	
IV	1 (3.5)	1 (5)	0 (0)	
*Lymphatic invasion*				
Absent	25 (86.2)	17 (85)	8 (89.9)	1
Present	4 (13.8)	3 (15)	1 (11.1)	
*Vascular invasion*				
Absent	14 (49.3)	9 (45)	5 (55.6)	0.7
Present	15 (51.7)	11 (55)	4 (44.4)	
*Pleural invasion*				
Absent	13 (44.8)	8 (40)	5 (55.6)	0.688
Present	16 (55.2)	12 (60)	4 (44.4)	

*PD-L1* programmed cell death ligand 1, *CT* chest computed tomography, *FDG* fluorodeoxyglucose, *PET/CT* positron emission tomography/CT

## Discussion

In this study, we retrospectively investigated the clinicopathological characteristics of patients with PPC who were surgically treated in our hospital. PPC is generally associated with a poor prognosis with a 5-year OS rate of 20–48% after surgical resection [3, 4, 8–10]. In the present study, we observed a favorable long-term survival rate of 60% compared with previous studies. This is presumably attributed to the absence of locally advanced cases, such as T3 tumors extending beyond parietal pleura, which were frequently observed in previous studies [4, 9, 11]. Tumor relapse tends to occur within one year of surgery [3, 4, 11, 12]. Similarly, 11 of 13 relapses were observed within 6 months of surgery, and most cases were detected as distant metastases. This finding might imply that identifying such an aggressive subgroup in this entity would lead to clarify appropriate candidates for neoadjuvant or adjuvant therapy in patients with curative resection of PPC. The strategy on adjuvant chemotherapy in the current study does not seem consistent, and no patients received pre- or postoperative immune checkpoint inhibitors (ICIs). These facts made it difficult to discuss implications for neoadjuvant or adjuvant therapy in PPC.

Several factors have been reported to be determinants of poor outcomes in patients with PPC, such as lymph node metastasis, tumor size, and pleural invasion [3, 4, 8–10, 13]. However, most studies were retrospective analyses with small sample sizes with the prognostic factors remaining unclear. In particular, the association between imaging findings and prognostic factors of PPC has been reported in only a few articles to date. The CT features of PPC are almost the same as those of NSCLCs, but the possibility of PPC might be suggested when a subpleural necrotic tumor is detected with peritumoral areas of ground-glass opacification and regional invasion to the chest wall or mediastinum [14]. A central low-attenuation area or cavity on CT was reported to have a poor prognosis and represents tumor necrosis on histopathological specimens [15]. Pathological massive coagulation necrosis indicates aggressive tumor growth, thereby contributing to poor prognosis [8]. On the contrary, air bronchogram on CT was suggested to be a favorable prognostic factor, which might reflect intact intratumoral bronchi without tumor invasion [16]. In addition to the cavity in the tumor, tumor size, and pleural invasion, our study showed for the first time that ringed FDG uptake on PET/CT was a significant prognostic factor of PPC. In 5 cases, the tumor had no cavity macroscopically or on CT, but revealed ringed FDG uptake on PET/CT (Fig. 1a–c). The ringed uptake supposedly indicates central necrosis, which is an aggressive feature that was later confirmed histologically (Fig. 1d). PET/CT might potentially detect central necrosis prior to cavity formation on CT and has important clinical implications for the prognosis of PPC.

PD-L1 on antigen-presenting cells or tumor cells, when interacting with PD-1 on T cells, negatively regulates T cell activation or immune response against tumor cells [17, 18]. Accordingly, PD-L1 expression in tumors is thought to affect tumor behavior and prognosis. PD-L1 is expressed more frequently in PPCs than in NSCLCs. In a previous report, PD-L1 was expressed in 75% of PPC cases [5] but was expressed in approximately 20% of NSCLC [19] or adenocarcinoma cases [20]. Similarly, in the present study, 69% of PPC cases expressed PD-L1. These studies, including the current one, used clone E1L3N and 5% as the cut-off of positivity. Younger patients [21] and parietal pleural invasion [12] were reportedly associated with higher PD-L1 expression in PPC patients. A meta-analysis of over 11,000 NSCLC patients revealed that higher expression of PD-L1 was associated with the male sex, smoking history, tumor size, and lymph node metastases but not age [22]. The current study showed that PD-L1 expression was associated with smoking history but not sex, age, or tumor size. Therefore, meta-analyses or multicenter prospective studies are necessary to define the correlation between PD-L1 expression and clinicopathological features. The association between PD-L1 and prognosis remains inconclusive, although several retrospective studies have focused on PPC. This is partly due to varieties of the clone of antibodies as well as cut-off values, both of which probably have a substantial influence [21, 22]. In addition, factors regarding whether or not surgical resection is indicated and whether or not ICI was available or is indicated would affect the analyses. The present study only included surgical cases with or without adjuvant chemotherapy other than ICI and did not demonstrate any prognostic impact for PD-L1 expression even though other cut-off values were applied (data not shown). More recently, next generation sequencing has revealed intratumoral heterogeneity in PD-L1 expression between epithelial and sarcomatoid components, in contrast to similarity in driver mutations [23], suggesting necessity of careful assessment of PD-L1 in each component to evaluate or predict the efficacy of ICI on PPC for future studies.

The current study has several limitations. First, our analysis was based on a very small number of patients from one institution because of the rarity of PPCs; hence, our findings are inconclusive. Second, the retrospective nature of this study may lead to the potential risk of several biases. Therefore, our results should be interpreted with consideration of these limitations, and multicenter prospective studies are needed to validate our findings.

## Conclusions

To the best of our knowledge, the present study showed for the first time that ringed FDG uptake on PET/CT was a significant prognostic factor of PPC, in addition to a cavity on CT and tumor size, which affected both DFS and OS. Pleural invasion affected DFS but not OS. PD-L1 expression did not have any impact on DFS or OS.

## Data Availability

The datasets used and/or analysed during the current study are available from the corresponding author on reasonable request.

## Abbreviations

PPC: Pulmonary pleomorphic carcinoma
WHO: World Health Organization
NSCLC: Non-small-cell lung cancer
ICI: Immune checkpoint inhibitor
PD-L1: Programmed cell death ligand 1
CT: Computed tomography
PET: Positron emission tomography
MRI: Magnetic resonance imaging
FDG: Fluorodeoxyglucose
PD-L1: Programmed cell death ligand 1
TPS: Tumor proportion score
OS: Overall survival
DFS: Disease-free survival
